# Artificial Intelligence in the Image-Guided Care of Atrial Fibrillation

**DOI:** 10.3390/life13091870

**Published:** 2023-09-05

**Authors:** Yiheng Lyu, Mohammed Bennamoun, Naeha Sharif, Gregory Y. H. Lip, Girish Dwivedi

**Affiliations:** 1 Department of Computer Science and Software Engineering, School of Physics, Mathematics and Computing, The University of Western Australia, Perth, WA 6009, Australia; yiheng.lyu@research.uwa.edu.au (Y.L.); mohammed.bennamoun@uwa.edu.au (M.B.);; 2 Harry Perkins Institute of Medical Research, The University of Western Australia, Perth, WA 6009, Australia; 3 Liverpool Centre for Cardiovascular Science, University of Liverpool, Liverpool L69 3BX, UK; 4 Liverpool John Moores University, Liverpool L3 5UX, UK; 5 Liverpool Heart and Chest Hospital, Liverpool L14 3PE, UK; 6 Danish Center for Health Services Research, Department of Clinical Medicine, Aalborg University, 9220 Aalborg, Denmark; 7 Department of Cardiology, Fiona Stanley Hospital, Perth, WA 6150, Australia; 8 Medical School, The University of Western Australia, Perth, WA 6009, Australia

**Keywords:** atrial fibrillation, artificial intelligence, machine learning, deep learning, echocardiography, computed tomography, magnetic resonance imaging

## Abstract

Atrial fibrillation arises mainly due to abnormalities in the cardiac conduction system and is associated with anatomical remodeling of the atria and the pulmonary veins. Cardiovascular imaging techniques, such as echocardiography, computed tomography, and magnetic resonance imaging, are crucial in the management of atrial fibrillation, as they not only provide anatomical context to evaluate structural alterations but also help in determining treatment strategies. However, interpreting these images requires significant human expertise. The potential of artificial intelligence in analyzing these images has been repeatedly suggested due to its ability to automate the process with precision comparable to human experts. This review summarizes the benefits of artificial intelligence in enhancing the clinical care of patients with atrial fibrillation through cardiovascular image analysis. It provides a detailed overview of the two most critical steps in image-guided AF management, namely, segmentation and classification. For segmentation, the state-of-the-art artificial intelligence methodologies and the factors influencing the segmentation performance are discussed. For classification, the applications of artificial intelligence in the diagnosis and prognosis of atrial fibrillation are provided. Finally, this review also scrutinizes the current challenges hindering the clinical applicability of these methods, with the aim of guiding future research toward more effective integration into clinical practice.

## 1. Introduction

Atrial fibrillation (AF) is the most common cardiac rhythm disorder. The prevalence of AF is high and has seen a significant surge over the past decades, with an estimated 33 million people worldwide suffering from this condition [1]. AF is associated with an increased risk of mortality and morbidity from dementia, heart failure (HF), and stroke [2]. The diagnosis of AF is typically made using electrocardiography (ECG), which records the heart’s electrical activity over time. The presence of AF is identified by the absence of P waves with irregular R-R intervals [3]. However, ECG has its limitations, such as the inability to provide spatial references for evaluating structural changes in the atria or visual guidance for invasive procedures.

In addition to ECG, cardiovascular imaging modalities such as echocardiography, computed tomography (CT), and magnetic resonance imaging (MRI) are often utilized to characterize AF [4]. The roles of cardiovascular imaging in the management of AF include, but are not limited to:

- Assessing structural changes in the heart, such as fibrosis tissue in the atria [5];
- Measuring imaging biomarkers, such as the volume of the left atrium (LA) [6,7];
- Offering visual guidance for invasive procedures for the treatment of AF, such as catheter ablation [8].

**Figure 1:**
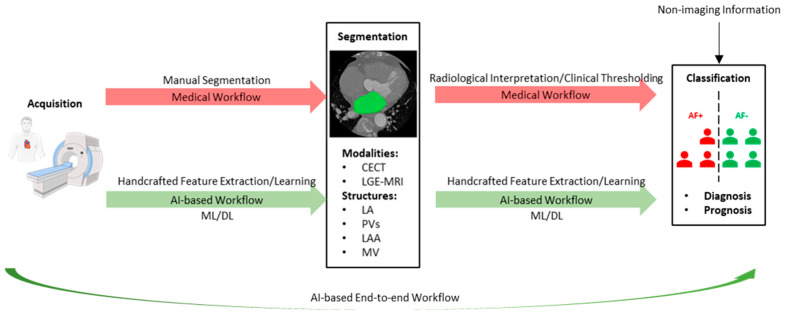
Steps involved in the image-guided care of atrial fibrillation. CECT—contrast−enhanced computed tomography, LGE—late gadolinium−enhanced, MRI—magnetic resonance imaging, LA—left atrium, PV—pulmonary vein, LAA—left atrial appendage, MV—mitral valve, AF+/−—patients with/without atrial fibrillation, AI—artificial intelligence, ML—machine learning, DL—deep learning.

Figure 1 illustrates the two most critical steps in image-guided AF care, namely, segmentation and classification. In the medical workflow, the segmentation step is manually performed by clinical experts. Manual segmentation of the structures of interests is labor-intensive and suffers from high intra-observer and inter-observer variability. Following the segmentation step, imaging biomarkers are measured through radiological interpretation and clinical thresholding, which are subsequently used in the classification step to support the diagnosis and prognosis of AF. In the medical workflow, classification is performed using statistical approaches, such as scoring systems [9]. However, such methods are based on sparse imaging biomarkers and non-imaging information, which may result in an oversimplification of the actual scenario. Hence, in the medical workflow, intra-observer and inter-observer variability reduces the accuracy and consistency of segmentation and classification, presenting significant challenges for the diagnosis and management of AF in terms of human resources and the reliability of results.

Recent breakthroughs in artificial intelligence (AI) have had a profound impact on the field of cardiovascular imaging [10], leading to substantial changes in image-guided AF care. As illustrated in Figure 1, in the AI-based workflow, segmentation and classification are carried out by deploying algorithms. Two forms of AI methodologies that can be employed for this purpose include classical machine learning (ML) and deep learning (DL). A key difference between the two lies in the type of input data they can process. Classical ML models require handcrafted features, for example, the volume of an anatomical structure. The handcrafted features used for an ML model are automatically extracted from images. This type of input data is considered structured data. In contrast, DL uses neural networks with multiple hidden layers to learn the features from the unstructured raw input data, such as images or videos, without requiring handcrafted feature extraction [11]. Hence, with the use of ML and DL algorithms, segmentation and classification can be performed with high accuracy, efficiency, and reproducibility. Furthermore, DL can identify complex patterns in imaging data, enabling direct diagnosis and prognosis of AF following image acquisition, constructing a highly accurate and efficient AI-based end-to-end workflow (Figure 1).

In this review, we explore the role of AI in image-guided AF care. We specifically focus on two AI-powered imaging tasks, namely, segmentation (Section 2) and classification (Section 3), as they have been the primary focus of research so far. Moreover, we discuss the future opportunities (Section 4) for further improvements in AI-assisted image-guided AF care.

## 2. Artificial Intelligence for Segmentation

Segmentation is the process of identifying and outlining structures of interest in raw images. The input for a segmentation task is raw images, and the output consists of segmentation maps for the targeted structures/objects. The structure of primary interest in patients with AF is the LA, as its volume has been associated with the prognosis of AF [6,7]. Furthermore, the LA is anatomically connected to the pulmonary veins (PVs), which have long been recognized as the main sources of the triggers of AF [12]. Other structures of interest include the substructures of the LA, such as the left atrial appendage (LAA) and the mitral valve (MV). Due to the complex cardiac anatomy, clinicians face challenges in using raw images for decision-making or treatment guidance. This makes segmentation a critical preliminary step in the clinical workflow.

**Figure 2:**
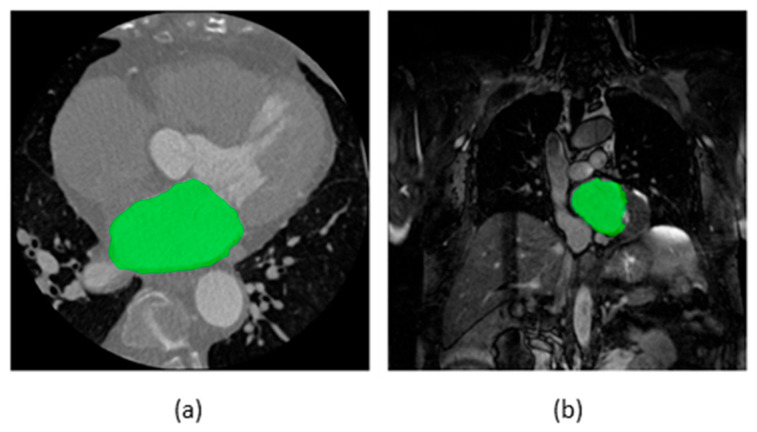
Segmentation maps of the left atrium superimposed on axial slices of (**a**) computed tomography and (**b**) magnetic resonance imaging scan. Source of scans: multi-modality whole-heart segmentation [21,22,23].

Figure 2 illustrates some examples of segmentation maps of the LA superimposed on raw images of CT and MRI. An AI-based segmentation model performs segmentation by categorizing each pixel of the input images as either belonging to the structure of interest (foreground/positive) or not (background/negative). Table 1 provides a summary of selected publications on AI-based segmentation methods for image-assisted AF care. Notably, the definitions of the structure of interest differ among the datasets used by different publications. For instance, in the 2018 LA segmentation challenge (LASC) dataset [13], currently the largest open-source dataset for LA segmentation on late gadolinium-enhanced MRI (LGE-MRI) in patients with AF, the structure of interest was defined as the pixels within the LA endocardial surface, including the MV and the LAA, as well as the extent of the PV sleeves. In the datasets used in other selected publications, the definitions of the structure of interest vary, including solely the LA [14,15] or various combinations of the LA and its substructures [16,17,18] on contrast-enhanced CT (CECT) or LGE-MRI. In addition, Jin et al. [19] proposed a model for the segmentation of the LAA on CECT, which is desirable for LAA occlusion procedures [20].

### 2.1. Methodologies

DL methods are employed for automated segmentation due to their ability to handle unstructured data as input. A convolutional neural network (CNN) is a specific type of DL method that is particularly adept at handling visual inputs, such as images [28]. CNN uses convolution operators to capture the relationships between adjacent pixels and has been used as the de facto method for medical image segmentation [29]. The methodologies of AI-based segmentation models include the architectures and building blocks (Section 2.1.1) of the CNNs, as well as the training process of the CNN-based segmentation models (Section 2.1.2).

#### 2.1.1. Architectures and Building Blocks

**Figure 3:**
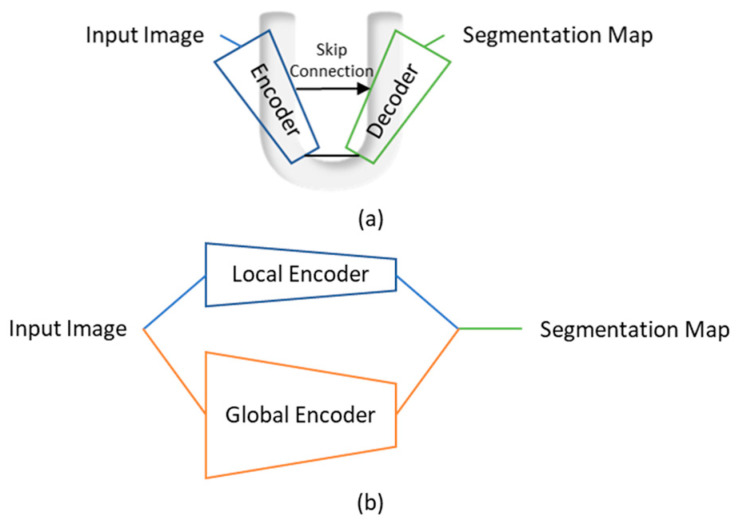
Typical architectures of medical image segmentation models. (**a**) U-net with a single-path encoder–decoder architecture. Various building blocks can be integrated with U-net to improve segmentation performance. (**b**) AtriaNet has a dual-path architecture with both a local and a global encoder. The local encoder performs feature learning on a smaller region, while the global encoder performs feature learning on a larger region.

Since its emergence in 2015, U-net [30] has been a popular architecture for medical image segmentation, and serves as the foundation for some of the state-of-the-art segmentation models [31,32,33]. U-net uses a single-path encoder–decoder architecture, forming a “U” shape, as illustrated in Figure 3a. The encoder consists of multiple convolution layers, which facilitate feature learning while decreasing image resolution. The decoder generates the output segmentation maps while restoring image resolution. Skip connections [34] are introduced between the encoder and decoder in order to allow the fine details learned in the encoder to contribute to the output segmentation maps. For LA segmentation, additional building blocks were integrated with the U-net architecture to improve segmentation performance. Table 2 provides a list of the important building blocks used with U-net architecture for LA segmentation. Convolutional long short-term memory (ConvLSTM) [35] layers were incorporated into the U-net [30] architecture to learn sequential information [16]. Batch normalization [36] layers were inserted for accelerating model training [15,17,26], while dropout [37] was adopted to prevent overfitting [15,26]. In addition, Grigoriadis et al. [18] adopted ResUnet++ [32], which integrated squeeze-and-excitation [38] blocks in the encoder and atrous spatial pyramidal pooling (ASPP) [39], as well as attention [40] blocks in the decoder.

While U-net has demonstrated its effectiveness in learning local features, its single-path architecture can hinder its ability to capture larger-scale features. Xiong et al. proposed AtriaNet [24], a model with a unique dual-path architecture for LA segmentation. As illustrated in Figure 3b, AtriaNet uses a local encoder and a global encoder for feature learning at local and global scales, both centralized on small image patches. The local encoder collects detailed geometric information about the LA for each pixel within its immediate vicinity. On the other hand, the global encoder gathers information on the position and overall structure of the LA. In order to reduce the number of parameters in the global encoder, max pooling was applied in the first layer of the global encoder so that the resolution of large input images could be decreased. The learned local and global features were merged to generate the output segmentation maps for the small image patches. Du et al. [25] also adopted the concept of a dual-path structure and multiscale feature learning and proposed a segmentation model integrating dual-path modules (DPMs) and multiscale context-aware modules (MSCMs) to facilitate multiscale feature learning.

U-net [30] was originally designed for two-dimensional (2D) medical image segmentation in a slice-by-slice manner. However, 2D segmentation from the axial slices can overlook the valuable three-dimensional (3D) information of the LA. Yang et al. [16] proposed a framework to utilize the 3D information, including the correlation between adjacent axial slices and multiview information. First, the framework adopted the U-net architecture, which used ConvLSTM [35] layers to connect the encoder and decoder. Second, the framework utilized dilated residual learning to learn features from the sagittal and coronal views. Methods for direct 3D LA segmentation [14,26,27] have also been proposed. Borra et al. [26] demonstrated that the 3D variant of U-net outperforms its 2D counterpart for LA segmentation. Liu et al. [27] designed their network with V-net [41], a 3D encoder–decoder architecture for volumetric medical image segmentation, as the backbone. Their design features a symmetric multilevel supervision (SML) structure, including auxiliary supervision branches added to both the encoder and the decoder, with convolution attention blocks inserted to connect the branches to the backbone. Furthermore, 3D LA segmentation was also widely adopted in the 2018 LASC, as it was used in 8 out of the 15 submitted CNN-based methods [13], including the winning model [42] that used 3D LA localization as a preliminary step.

In summary, U-net [30] with its encoder–decoder architecture has been serving as the foundation for state-of-the-art models for LA segmentation. Architectures with unique designs, as well as building blocks, were used to improve segmentation performance, primarily in terms of multiscale feature learning and utilizing the 3D information contained in the volumetric images. In the next section, we explore how the state-of-the-art models were trained to perform segmentation.

#### 2.1.2. Training Segmentation Models

Training segmentation models is as important as designing the architecture and building blocks. A segmentation model is trained to learn the mapping from the input images to the output segmentation maps. Supervised learning is the dominant approach in the development of AI models for segmentation, as indicated by the number of publications [43]. This approach requires labeled data that include both the input images and the corresponding output segmentation maps, known as the ground truth. In the context of supervised learning, a segmentation model is trained by minimizing a loss function, which measures the deviation of the model output from the ground truth segmentation maps, thereby optimizing the model’s parameters. Two types of loss functions are widely used for the training of segmentation models. The first type refers to pixel-wise loss functions, including cross-entropy loss and mean squared error loss. These losses penalize the deviation of the model output from the ground truth at the pixel level. While pixel-wise loss functions are straightforward to implement, they can struggle with class imbalance when segmenting specific anatomical structures. A class imbalance arises when the volume of the structure of interest occupies a significantly smaller volume than the overall image, resulting in a disproportionate number of positive pixels compared to negative ones. For example, the volume of LA in patients with AF normally lies within the range of 90–180 mL [15]. This range of LA volume is significantly lower than the volume of the field of view of a routine cardiac CT or MRI scan, which is approximately 7000–8000 mL [13]. This huge class imbalance might cause a pixel-wise loss function to be insensitive to segmentation errors that are insignificant to the volume of the field of view yet significant to the LA volume.

**Figure 4:**
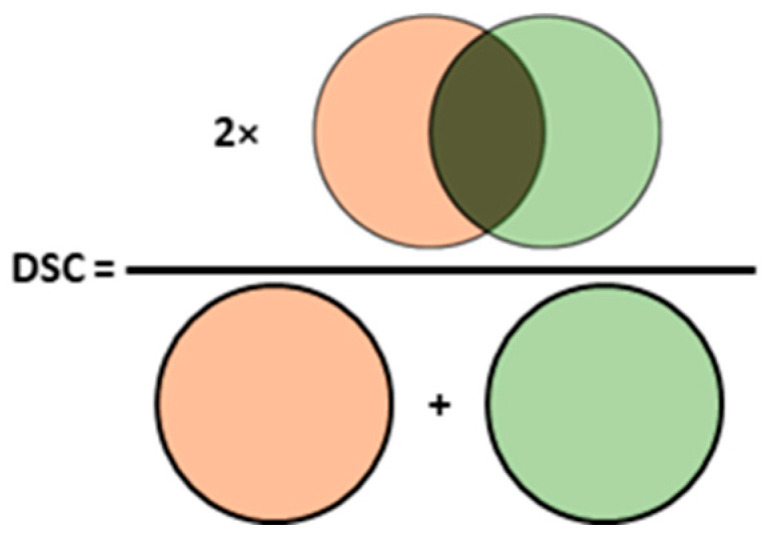
Dice similarity coefficient is given by twice the overlap between the output and the ground truth segmentation maps, divided by the sum of the two segmentation maps.

One strategy to address the class imbalance during the training process of a segmentation model is to use weighted pixel-wise loss functions. Alternatively, a second type of loss function can be used, which penalizes the deviation of the model output from the ground truth at the structural level. The most widely used structure-wise loss function is Dice loss, which is defined using the Dice similarity coefficient (DSC), as illustrated in Figure 4. DSC is a widely used metric for evaluating the performance of segmentation models. A greater DSC indicates a higher level of agreement between the model output and the ground truth segmentation, reflecting better overall model performance. There are also hybrid loss functions that combine pixel-wise and structure-wise loss functions to guide the training of segmentation models [14,19,25,27]. Specifically, Liu et al. [27] proposed a loss function guided by the segmentation uncertainty, which was measured by the Jensen–Shannon divergence between the predictions from the SML branches. The final hybrid loss function combined the integration of a cross-entropy loss calibrated by the Jensen–Shannon divergence and a Dice loss of both the SML branches.

Enhancing the generalizability of a segmentation model may necessitate pre-processing the raw images prior to feeding them into the model. Commonly used techniques for pre-processing include image histogram equalization, image intensity normalization, and the use of filters for denoising. nnU-net [33] offers an automated solution for such pre-processing techniques. Jin et al. [19] adopted a unique strategy for pre-processing that involved multiscale retinex with color restoration [44] for image enhancement, followed by the conversion of gray-level images to pseudo-color images, which improves the resolution of local feature learning. Another pre-step frequently required for LA segmentation is the detection or localization of the LA. LA detection can be useful for 2D LA segmentation and involves the process of determining whether the LA is present on a 2D slice. Grigoriadis et al. [18] and Abdulkareem et al. [15] performed LA detection manually and using a DL-based classification model, respectively. Slices without the presence of the LA were excluded before LA segmentation. LA localization, on the other hand, refers to the process of defining a bounding box around the LA. There are various ways to perform LA localization, such as defining manual fiducial points [19] or using Otsu’s algorithm [26]. DL-based methods can also be used to automate localization, as demonstrated by the top-performing model [42] of the 2018 LASC [13].

Supervised learning can be hindered by the scarcity of labeled data. Data augmentation is a technique used to address this issue by generating new samples from the existing ones, thereby expanding the training set. For LA segmentation, data augmentation techniques include elastic deformations, affine transformations, and warping [45]; the addition of Gaussian noise and changing contrast via power law transformation [17]; and random cropping [27] and intensity normalization [15]. An alternative approach to address the issue of scarce labeled datasets is active learning [46], which was adopted by Cho et al. [14] for LA segmentation using a human-in-the-loop strategy. This approach starts by training the model on a small, labeled dataset and then gradually feeding the model subsets of unlabeled samples in several stages. After each step, human experts modify the model output, which is then combined with the previous training set to retrain the model.

In summary, structure-wise or hybrid loss functions can be used to address the class imbalance when solely pixel-wise loss functions are used to guide the training of LA segmentation models. Common preliminary steps of LA segmentation include pre-processing, which improves the model generalizability, as well as LA detection and localization, which reduce computational expense. To overcome the challenge of scarce labeled datasets, data augmentation, and active learning can be adopted. In the next section, we explore how the architectures, building blocks, and training approaches influence the performance of the segmentation models.

### 2.2. Performance of Segmentation Models

The performance of a segmentation model has a direct impact on the subsequent procedures and eventually influences the quality of clinical care. To assess the performance of a segmentation model, various metrics are used to quantitatively evaluate the disagreement between the output of the AI model and the ground truth, which is established by human experts. These metrics can be broadly categorized into three types, namely, pixel-wise metrics, similarity-based metrics, and metrics based on surface distance. Pixel-wise evaluation metrics, including pixel-wise accuracy, precision, sensitivity, and specificity, are frequently used and simple to compute. However, the use of pixel-wise accuracy and specificity suffers from class imbalance. Specifically, publications [16,17,24,26] that reported pixel-wise accuracy and specificity achieved >0.995 in both of these metrics, resulting from the reliable exclusion of the background from the output segmentation maps. In contrast, pixel-wise precision and sensitivity show the capabilities of the models to include the foreground in the output segmentation maps and suffer less from class imbalance.

Similarity-based metrics are measured by the volume overlap between the output segmentation maps generated by the models and the ground truth segmentation maps. Besides DSC (Figure 4), which is frequently used for a comparison of the performance of segmentation models [13,43], the Jaccard similarity coefficient (JSC), which is computed as the intersection over the union of two volumes, can also be used. Both the pixel-wise and the similarity-based evaluation metrics lie within the range of [0, 1], and an increase in the metric indicates an increase in segmentation performance.

Evaluation metrics based on surface distance evaluate the geometrical characteristics of the output segmentation maps. These include Hausdorff distance (HD) and the average surface distance (ASD), which are defined as the maximum and the mean local distance, respectively. The local distance is defined as the minimum distance between a point on the surface of the output to the ground truth segmentation map, for all the points on the surface of the output segmentation maps. The evaluation metrics based on surface distance fall in the range of [0, +∞), and a decrease in the metric indicates an increase in segmentation performance. Notably, unlike other evaluation metrics, which measure the global segmentation performance, HD is highly sensitive to local segmentation errors. Additionally, metrics for clinically significant measurements, such as the diameter [24] and the volume [14,15,26] of the LA, can also be used for evaluating the performance of segmentation models.

Four of the papers reviewed [24,25,26,27] made use of the 2018 LASC dataset [13]. Among the four papers, Xiong et al. [24] had access to the 54 labeled scans in the testing dataset of the 2018 LASC dataset [13] and, thus, is not comparable to the other three papers. Having proposed AtriaNet, Xiong et al. [24] achieved 0.940 and 0.942 in DSC for segmentation of the LA endocardium and epicardium, respectively. The other three papers only had access to and made use of the training dataset of the 2018 LASC dataset [13] and, hence, are comparable. Table 3 provides the performance, in terms of DSC and HD, achieved in these three papers. Du et al. [25] applied a 2D segmentation model with DPMs, MSCMs, gated bidirectional message passing modules (GBMPMs), and deep supervision mechanisms, achieving top performance in terms of DSC. Borra et al. [26] used variants of U-net [30] that included batch normalization [36] layers and performed both 2D and 3D segmentation. The 3D segmentation outperformed its 2D counterpart in terms of both DSC and HD and achieved top performance among the three publications in terms of HD. Liu et al. [27] proposed a unique methodology, using a V-net [41] architecture with an SML structure and trained with an uncertainty-guided loss function for 3D segmentation.

Because of the various datasets and definitions of labels used in other reviewed papers, their segmentation performance is not directly comparable. Instead, we discuss the key factors that influence the performance of the segmentation models.

Post-processing is a step that uses established knowledge to modify the output segmentation maps generated by the segmentation model so that the segmentation performance can be improved. General post-processing operations used include applying a Gaussian filter or selecting only the largest connected tissue in 3D to represent the final LA segmentation. Interestingly, Borra et al. [26] reported that the use of 3D LA segmentation reduces the need for post-processing by 10% when compared to its 2D counterpart, demonstrating the potential superiority of 3D segmentation. Jin et al. [19] used a 3D conditional random field (CRF) [47] as a post-processing technique to improve the reconstructed 3D LAA volume after 2D LAA segmentation, resulting in a DSC of 0.9476. By exploiting the 3D spatial relationship between adjacent axial slices, their method corrected erroneous outputs with isolated regions or gaps in the 2D output segmentation maps.

Using data augmentation and active learning to address the issue of scarce labeled datasets improves segmentation performance. Xiong et al. [24] discovered that by a using data augmentation technique that warps 50% of the initial data, the performance of the model was enhanced by 0.005 in terms of DSC. Cho et al. [14] used active learning for LA segmentation with an initially small, labeled dataset. An increase in DSC was seen after each step with human intervention, with the DSC improved from 0.85 to 0.89 to 0.90.

**Figure 5:**
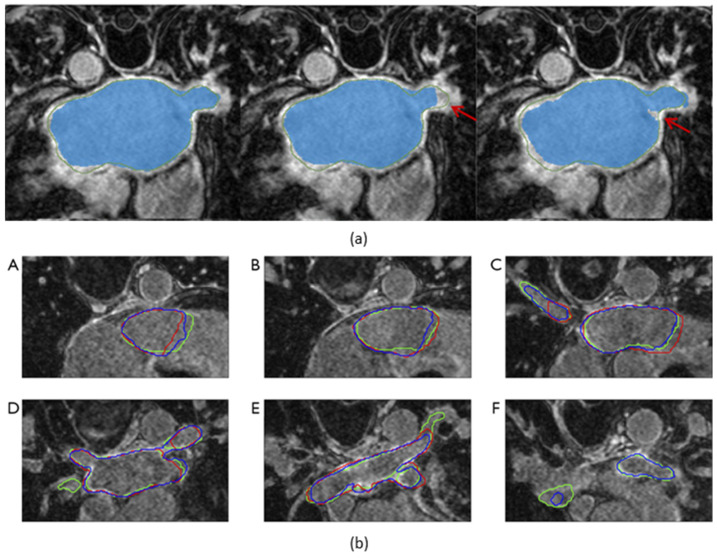
Visualization of the output segmentation maps superimposed on axial slices of late gadolinium-enhanced magnetic resonance imaging scans. (**a**) Comparison of the output segmentation maps. The green contours and the blue masks represent the ground truth and the model’s output segmentation maps, respectively. From left to right: the model with both the symmetric multilevel supervision (SML) structure and the uncertainty-guided loss function, the model with only the SML structure, and the model with only an auxiliary supervision branch added to the decoder. Red arrows point out disagreements between the model output and the ground truth. (**b**) This shows a decline in segmentation accuracy in the vicinity of the pulmonary veins. (**A**–**F**): axial slices 15%, 25%, 40%, 60%, 75%, and 85% along the longitudinal axis of the left atrium. The green, red, and blue contours represent the segmentation maps of the ground truth, 2-dimensional segmentation model, and 3-dimensional segmentation model, respectively. The 2-dimensional segmentation model completely failed on the slice 85% along the axis. Figure source: (**a**) [27], (**b**) [26].

The impact of individual components of a proposed method can be evaluated by systematically removing these components and observing the impact on the model’s performance. Du et al. [25] enhanced its architecture by gradually introducing DPMs, MSCMs, GBMPMs, and a deep supervision mechanism, resulting in an improved DSC with each addition. Liu et al. [27] compared their proposed model with two other segmentation models. The first model had only an auxiliary supervision branch added to the decoder, while the second model had an SML structure but lacked an uncertainty-guided loss function. Their results indicated that incorporating an auxiliary supervision branch to the encoder improved both DSC and HD, while additionally including an uncertainty-guided loss function further improved the segmentation of the fuzzy surface of the LA, as illustrated in Figure 5a, leading to a reduction in HD.

While high performance in LA segmentation has been demonstrated by state-of-the-art segmentation models, suboptimal segmentation performance has been reported by multiple papers [17,25,26] in regions containing substructures of the LA. Specifically, Razeghi et al. [17] and Borra et al. [26] reported local segmentation performance in regions containing the PVs and the MV. Razeghi et al. [17] conducted 2D segmentation of the LA, MV, and PVs separately. While LA segmentation resulted in a DSC of 0.91 ± 0.02, which is consistent with the other state-of-the-art segmentation models, the segmentation of the PVs and the MV resulted in a DSC of 0.61 ± 0.08 and 0.73 ± 0.08, respectively, showing a decline in overall segmentation performance. Similarly, Borra et al. [26] examined the segmentation performance of the LA along its longitudinal axis, which was divided into three sub-volumes: adjacent to the MV, containing the LA body, and encompassing the PVs. While DSC remained relatively stable in the middle sub-volumes containing the LA body, a significant decrease was observed in the sub-volumes adjacent to the MV and encompassing the PVs. In these sub-volumes, 2D segmentation exhibited a greater decrease in performance compared to 3D segmentation, with a notably low DSC observed in the sub-volume containing the PVs, as shown in Figure 5b. Furthermore, Liu et al. [27] demonstrated that using V-net [41] resulted in high segmentation uncertainty in the regions with the PVs, while the addition of the SML structure as well as the uncertainty-guided loss function reduced the segmentation uncertainty. The PVs play a critical role in the onset of AF [12], but their shapes are highly complex and vary significantly between patients. In patients selected to receive catheter ablation, the most frequently practiced technique is PV isolation, which aims to electrically isolate the triggers in the PVs from the LA [8]. For the safety and effectiveness of PV isolation, it is crucial that the PVs can be segmented accurately. Future research should explore more accurate segmentation techniques to address the challenging shape of the PVs.

Finally, we found out that although not directly comparable, the segmentation of the LA and its substructures, performed on LGE-MRI, resulted in higher segmentation performance than segmentation performed on CECT. Specifically, the segmentation of the LA, including the PVs, on LGE-MRI typically resulted in a mean DSC over 0.9 [24,25,26,27], with the sole exception of the publication by Yang et al. [16], which achieved a mean DSC of 0.897 ± 0.053. In contrast, the segmentation of the LA, including the PVs, on CECT resulted in a mean DSC of 0.80 [18]. Similarly, when focusing on the segmentation of solely the LA, the mean DSCs were 0.91 and 0.885 on LGE-MRI [17] and CECT [15], respectively, with similar segmentation models based on variants of U-net [30]. Because of the differences in the physics of image acquisition, LGE-MRI provides higher contrast when imaging the heart, resulting in higher image quality in terms of the signal-to-noise ratio, which is more desirable for AI-based segmentation models [13].

In summary, pixel-wise metrics, similarity-based metrics, and metrics based on surface distance can be used for the evaluation of LA segmentation models. Post-processing, data augmentation, and active learning techniques, as well as unique designs in model architecture and loss function, improve segmentation performance. Segmentation performance is also influenced by the type of structures contained in the regions of interest, as well as the imaging modalities. Relatively high performance has been achieved for the segmentation of the LA body on LGE-MRI. However, accurate segmentation of the substructures of the LA, especially the PVs, remains a challenge. In addition, more robust segmentation models need to be developed so that the performance of segmentation performed on CECT scans can approach the performance achieved on LGE-MRI scans.

## 3. Artificial Intelligence for Classification

**Figure 6:**
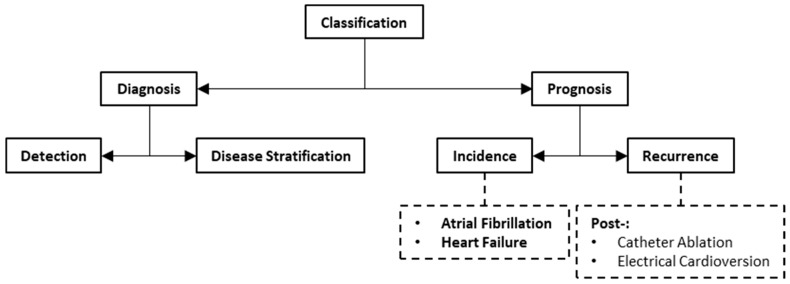
Taxonomy of artificial intelligence methods for classification.

Classification involves assigning samples to one or more predefined categories based on some observed characteristics or features. These samples could be a group of patients, or the acquired images of the group of patients. The categories could represent the presence or absence of a disease, or different subtypes of a disease. A taxonomy of AI-based classification for AF is illustrated in Figure 6. We can broadly categorize classification into the diagnosis and prognosis of AF. Table 4 provides a summary of publications on AI-based classification methods for image-assisted care of AF.

### 3.1. Feature Engineering

When ML methods are used for classification, handcrafted features are extracted and selected from images as well as non-imaging information in a process named feature engineering. The feature engineering process is a crucial preliminary step for the image-guided characterization of AF. Handcrafted features can be categorized into four types: imaging biomarkers, radiomic features, biophysical modeling features, and non-imaging features. Imaging biomarkers are clinically recognized features that can serve as indicators of cardiac function or physiology [48]. These imaging biomarkers are typically extracted from the segmentation of the structures of interest. For patients with AF, an example of an imaging biomarker is the volume of the LA, which has been identified as a predictor for the successful restoration of sinus rhythm through PV isolation [6] as well as for post-ablation AF recurrence [7]. The extraction of imaging biomarkers has been significantly enhanced by AI-based segmentation methods [13].

**Table 4:** Summary of publications on artificial intelligence for classification included.

Publication (Year)	Classification Task ^1^	Imaging Modality	Evaluation Metrics	AUC ^2^	Highlights ^3^
Shade et al. (2020) [49]	Recurrent AF prediction AF+ (n = 12) AF− (n = 20)	LGE-MRI	AUC, sensitivity, specificity	0.82	Quadratic discriminant analysis with radiomic and biophysical modeling features. Contribution of biophysical modeling features is significantly greater than radiomic features. Using biophysical modeling features enables accurate recurrent AF prediction even with a small dataset.
Vinter et al. (2020) [50]	Electrical cardioversion success prediction	TTE	AUC	0.60 (0.54–0.67)	Logistic regression with imaging biomarkers and non-imaging features. Sex-specific classification models achieved suboptimal performance in electrical cardioversion success prediction.
	Women Success (n = 149) Failure (n = 183)				
	Men Success (n = 394) Failure (n = 396)			0.59 (0.55–0.63)	
Liu et al. (2020) [51]	AF Trigger origin stratification ^4^ Only PV trigger (n = 298) With non-PV trigger (n = 60)	CECT	AUC, accuracy, sensitivity, specificity	0.88 ± 0.07	ResNet34-based model identifies patients with non-PV triggers of AF from axial CECT slices. Decision making of the model is based on morphology of the LA, right atrium (RA), and PVs.
Zhou et al. (2020) [52]	Incident AF prediction AF+ (n = 653) AF− (n = 3656)	TTE	AUC, area under the precision-recall curve	0.787 (0.782–0.792)	Logistic regression with imaging biomarkers and non-imaging features. Age is the sole predictive variable for incident AF prediction in oncology patients. Time-split data ensures model generalizability.
Hwang et al. (2020) [53]	Recurrent AF prediction AF+ (n = 163) AF− (n = 163)	TTE	AUC, accuracy, sensitivity, specificity	0.861	CNN-based model outperforms ML model in prediction of post-ablation AF recurrence when using curved M-mode images of global strain and global strain rate generated from TTE.
Firouznia et al. (2021) [54]	Recurrent AF prediction AF+ (n = 88) AF− (n = 115)	CECT	AUC	0.87 (0.82–0.93)	Random forest with radiomic and non-imaging features. AF induced anatomical remodeling of the LA and PVs is associated with increased roughness in the morphology of these structures.
Matsumoto et al. (2022) [55]	AF detection ^5^ AF+ (n = 1724) AF− (n = 12144)	Radiography	AUC, accuracy, precision, negative predictive value, sensitivity, specificity	0.80 (0.76–0.84)	Classification model based on EfficientNet identifies patients with AF from chest radiography. Regions that received more attention are the LA (the most) and the RA (the 2nd most) regions.
Zhang et al. (2022) [56]	AF detection ^6^	CECT	AUC, accuracy, sensitivity, specificity	0.92 (0.84–1.00)	Random forest with radiomic features. ML classification models identify patients with AF from EAT on chest CECT and CT.
	n = 200				
	n = 300	CT		0.85 (0.77–0.92)	
Roney et al. (2022) [57]	Recurrent AF prediction AF+ (n = 34) AF− (n = 65)	LGE-MRI	AUC, accuracy, precision, sensitivity	0.85 ± 0.09	SVM with PCA model, with imaging biomarker, biophysical modeling, and non-imaging features. ML classification model enables personalized prognosis of AF after catheter ablation
Yang et al. (2022) [58]	AF subtype stratification PAF (n = 207) PeAF (n = 107)	CECT	AUC, accuracy, sensitivity, specificity	0.853 (0.755–0.951)	A nomogram integrating imaging biomarkers and radiomic features.
	Recurrent AF prediction AF+ (n = 79)AF− (n = 235)			0.793 (0.654–0.931)	Random forest with radiomic features. Radiomic features based on first order and texture correlate with the inflammatory tissue in the atria.
Dykstra et al. (2022) [59]	Incident AF prediction AF+ (n = 314) AF− (n = 7325)	LGE-MRI	AUC	0.80/0.79/0.78 ^7^	Random survival forests with imaging biomarkers and non-imaging features. Time-dependent risk prediction of incident AF in patients with cardiovascular diseases.
Hamatani et al. (2022) [60]	Incident HF prediction HF+ (n = 606) HF− (n = 3790)	TTE Radiography	AUC, accuracy, sensitivity, specificity	0.75 ± 0.01	Random forest with imaging biomarkers and non-imaging features. Importance of imaging biomarkers extracted from TTE for incident HF in patients with AF.
Pujadas et al. (2022) [61]	Incident AF prediction AF+ (n = 193) AF− (n = 193)	MRI	AUC, accuracy, sensitivity, specificity	0.76 ± 0.07	SVM with radiomic and non-imaging features. Radiomic features based on shape and texture correlate with chamber enlargement and hypertrophy predispose AF, adverse changes in tissue composition of the myocardium, as well as LV diastolic dysfunction.

^1^ The classification task and the number of samples in each class of each publication. Classification was performed on patient level unless otherwise stated. ^2^ AUC achieved in the top performing model of each publication. The original values of AUC reported in the publications are provided. The AUCs are provided as AUC, AUC (95% confidence interval), or AUC ± standard deviation. The AUCs are not directly comparable since different datasets were used for different classification tasks. ^3^ Highlights provide (1) the top-performing model and the categories of features selected for ML-based model; (2) the key findings. ^4^ Original classification was performed on slice level. Patient-level classification was acquired by aggregating all slice-level decisions for each patient. ^5^ Classification was performed on scan level. ^6^ Number of patients in each class (AF+/AF−) not given. ^7^ Time-dependent AUC at 1 year/2 years/3 years.

**Figure 7:**
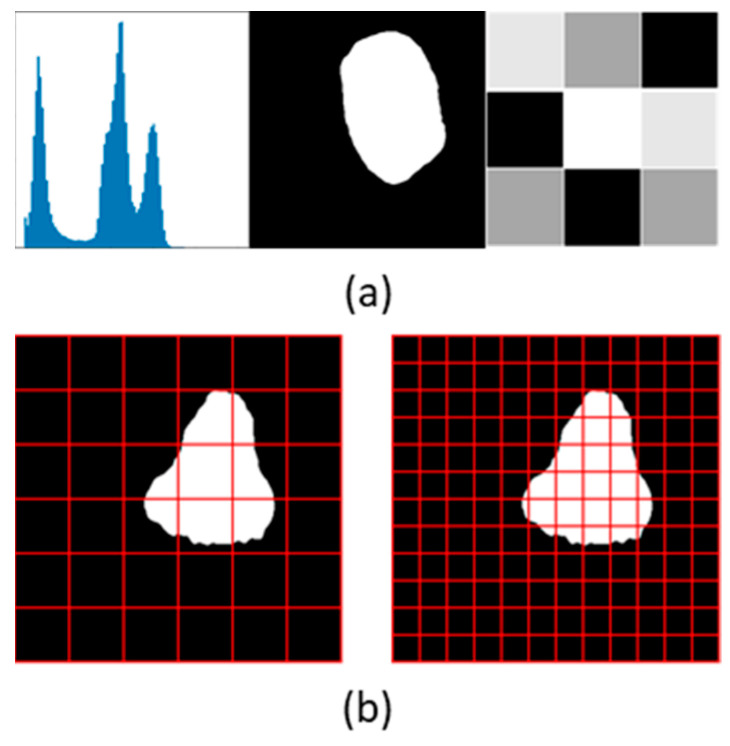
Visual illustration of radiomic features. (**a**) From left to right: radiomic features based on first-order, shape, and texture features. (**b**) A two-dimensional representation of a radiomic feature based on the fractal dimension of the structure of interest. This feature can be calculated as the difference in the number of cells of various sizes to cover the entire boundary of the structure of interest on a two-dimensional slice.

Radiomic features are high-level features that are typically not clinically recognized, nor can they be identified or evaluated with the naked eye. They are quantitative features that can be extracted from images through mathematical operations. The process of automated extraction of a large number of radiomic features is known as radiomics [62]. Radiomic features can be classified as first-order features, shape features, and texture features, as illustrated in Figure 7a. First-order features are based on the image histogram, shape features are based on the geometry of the structures studied, and texture features are based on the spatial distribution of the pixels [61]. Standardized definitions and validated reference values have been provided for a set of radiomic features [63], which can be extracted using open-source platforms, such as PyRadiomics [64] and QMaZda [65]. Radiomic features can be based on the fractal dimension of object structures, which provides a quantitative measure of their roughness [66]. An example of such a feature is the variation in the ratio of fractal dimension as the image resolution decreases, which is determined by the number of cells of different sizes needed to cover the boundary of the structure, as illustrated in Figure 7b.

In addition to imaging biomarkers and radiomic features, which can be extracted from raw images, the established knowledge of cardiovascular anatomy and electrophysiology (EP) can be integrated into biophysical modeling of the LA, which can be constructed from LGE-MRI [67,68]. Examples of features extracted from biophysical modeling include the number of reentrant drivers and macroreentrant atrial tachycardias observed within N most predictive anatomic regions [49] and dominant frequency measured 2 s post-ablation for various simulation set-ups [57]. Open-source platforms, such as openCARP [69], support simulations of AF, from which features can be extracted. Furthermore, non-imaging features extracted from electronic health records, laboratory tests, and patient health questionnaires also hold significance and can play a crucial role as complementary variables in classification tasks.

The number of features extracted from images can be large, especially in the case of radiomic features, where the count can exceed hundreds [62]. Ensuring effective feature selection is critical to avoid the curse of dimensionality. It helps reduce computational complexity, minimize the generalization error, and enhance the clinical explainability of the model [61]. Reproducibility is a vital aspect to consider when selecting radiomic features extracted from CT [70] or MRI [71]. The intraclass correlation coefficient with a cut-off value of 0.8 is commonly used for reproducibility tests. In addition, mutual information with a cut-off value of 0.05 [58] can be used to test the independency of radiomic features. A few methods, such as sequential feature forward selection [72], SHapley Additive exPlanations [73], and Boruta [74], can be used for selecting the most discriminative features, i.e., features that have the highest statistical significance based on their P-values. Alternatively, features that exhibit a strong correlation with the classification task can be selected based on clinical expertise [50].

In summary, feature engineering is a crucial step in classification in image-guided care of AF. Handcrafted features, including imaging biomarkers, radiomic features, biophysical modeling features, and non-imaging features, provide valuable information about cardiac function and structure, as well as patient history. Effective feature selection is essential to manage a large number of features, improve computational efficiency, and enhance clinical interpretability. In the next section, we explore the application of AI for the diagnosis of AF, including the use of ML models that require feature engineering, as well as the use of end-to-end DL models.

### 3.2. Artificial Intelligence for Diagnosis

Diagnosis includes detecting the presence of a disease and disease stratification. Matsumoto et al. [55] proposed an end-to-end approach for detecting AF in chest radiography. In their study, they used EfficientNet [75], a highly efficient and accurate CNN model. To facilitate feature learning, they leveraged a large dataset consisting of 7000 patients and 13,000 2D radiographs and achieved an area under the curve (AUC) of 0.80 (95% confidence interval (CI), 0.76–0.84). The relationship between obesity and an increased risk of AF [76] has been established, and research suggests that epicardial adipose tissue (EAT) significantly contributes to the development of AF substrates [77]. In a study conducted by Zhang et al. [56], EAT was investigated using CECT and non-enhanced CT. Through the utilization of radiomic features as inputs for random forest models, they yielded impressive results in AF detection, with AUCs of 0.92 (95% CI, 0.84–1.00) and 0.85 (95% CI, 0.77–0.92) for CECT and non-enhanced CT, respectively.

Based on the presentation, duration, and spontaneous termination of AF episodes, patients with AF can be stratified into having paroxysmal AF (PAF) or persistent AF (PeAF). In a study by Yang et al. [58], ML models were developed to distinguish between patients with PAF and PeAF based on EAT derived from CECT, whereby the most effective model was a nomogram that integrated imaging biomarkers and radiomic features, with an AUC of 0.853 (95% CI, 0.755–0.951). The imaging biomarkers included the volume of the LA, the volume of EAT, and the volume of EAT surrounding the LA. This integrated approach demonstrated superior performance in distinguishing between PAF and PeAF subtypes.

Patients diagnosed with AF can also be stratified based on the origin of triggers, distinguishing between those with only PV triggers and those with non-PV triggers. Prognosis and optimal treatment strategies vary depending on the absence/presence of non-PV triggers. For patients with only PV triggers, PV isolation is the preferred strategy for rhythm control; however, for patients with non-PV triggers of AF, receiving PV isolation as the sole strategy would likely lead to AF recurrence. Liu et al. [51] proposed a DL-based method to differentiate between patients with only PV triggers and those with non-PV triggers of AF. They employed a 34-layer residual network [34] to perform 2D image classification on axial slices of CECT. Their approach improved the classification performance by aggregating the decisions of all axial slices for a patient (AUC 0.88 ± 0.07), instead of performing slice-wise classification (AUC 0.82 ± 0.01).

AF can often go undetected until an adverse event occurs, such as a stroke. Although AF screening can facilitate early diagnosis, current clinical guidelines lack sufficient evidence to support the potential health benefits associated with ECG-based AF screening [78,79]. Chest radiography and CT are imaging modalities commonly used for screening for lung cancer and pulmonary diseases [80]. Because of the availability of datasets in chest radiography [55] and CT [80], AI can help screen for AF using chest scans. Specifically, novel DL-based methods [51,55] enabled the diagnosis of the abnormality of cardiac electrical activity from a 2D visualization of the cardiac anatomy.

In summary, researchers have explored AI-powered approaches for the diagnosis of AF, including AF detection and subtype stratification. When using ML models, EAT is an important source for extracting handcrafted features. With the development of DL algorithms, there is a potential promising feature of screening for AF using chest scans, as a complementary strategy to ECG-based methods for the diagnosis of AF.

### 3.3. Artificial Intelligence for Prognosis

Prediction models for incident AF have been developed in patients at risk of AF [52,59], as well as in the general population [61]. Cardiotoxicity induced by cancer therapy [81] poses a risk for cancer survivors, who may develop AF [82]. For example, Zhou et al. [52] developed ML models to predict incident AF in cancer survivors and achieved an AUC of 0.787 (95% CI, 0.782–0.792). They used time-split data: the patients who received treatments for cancer before or after a specific date were assigned to datasets for training and testing, respectively. This approach ensures independence between the training and testing sets, enhancing the generalizability of the models. Similarly, Dykstra et al. [59] developed ML models to predict incident AF in more than 7000 patients with other cardiovascular diseases, who were also at an elevated risk of developing AF. Both imaging biomarkers and non-imaging features were used in the study. The top-performing model, a random survival forest, incorporated several imaging biomarkers including the volume of the LA, the end-diastolic and end-systolic volume of the left ventricle (LV) and right ventricle, the mass of the LV, and all indexed to body surface area. Additionally, left ventricular ejection fraction (LVEF), significant valve heart disease, LV cardiac output, and bicuspid aortic valve were also included in the model. This model demonstrated the ability to predict incident AF with time-dependent AUCs of 0.80, 0.79, and 0.78 at 1, 2, and 3 years after LGE-MRI acquisition, respectively. Pujadas et al. [61] predicted incident AF in the participants of the UK Biobank imaging enhancement [83]. Using radiomic and non-imaging features with a support vector machine (SVM) model, an AUC of 0.76 ± 0.07 was achieved. Moreover, Pujadas et al. [61] found the information contained in the imaging biomarkers and the radiomic features to be correlated, as both of these two types of features contain information on the anatomic characteristics of the imaged patients. Specifically, a strong correlation was observed between the imaging biomarkers and the radiomic features related to size, the local uniformity, and shape [61]. This finding potentially suggests that imaging biomarkers are the least important category of features when radiomic features are used to predict incident AF.

HF is one of the complications of AF, and Hamatani et al. [60] developed a prediction model for incident HF in patients with AF. Imaging biomarkers and non-imaging features were extracted from the Fushimi AF Registry [84], which consists of a cohort of more than 4000 patients. The top-performing model, based on a random forest algorithm, incorporates various imaging biomarkers including cardiothoracic ratio extracted from chest radiography, as well as LVEF, left ventricular end-systolic diameter, and left ventricular asynergy extracted from transthoracic echocardiography (TTE). Comparing the model proposed by Hamatani et al. [60] to the renowned Framingham HF risk model [85], it demonstrated significantly superior performance in terms of AUC (0.75 vs. 0.67), indicating improved predictive accuracy for incident HF in patients with AF.

The prediction of AF recurrence is associated with rhythm control procedures in patients diagnosed with AF [8]. AI has been applied to predict both the procedural success as well as the postprocedural recurrence of AF. Electrical cardioversion is a non-invasive procedure used for rhythm control in patients with AF. For example, Vinter et al. [50] developed a gender-specific model to predict the success of electrical cardioversion. Imaging biomarkers, including LVEF and the diameter of the LA, along with non-imaging features, were used to develop ML models for women and men, but neither of the models achieved satisfactory performance. The top-performing models for women and men yielded AUCs of 0.60 (95% CI, 0.54–0.67) and 0.59 (95% CI, 0.55–0.63), respectively [50]. It remains unclear whether the relevance of the extracted features was low or if the procedural outcome of the electrical cardioversion is inherently unpredictable.

Compared to the model that predicts the success of electrical cardioversion, models that predict the postprocedural recurrence of AF have been shown to achieve much higher performance. The recurrence of AF after catheter ablation is driven by a complex interaction of various factors, and the prediction of AF recurrence is desirable for postprocedural risk assessment. Biophysical modeling of the LA can be constructed by integrating established knowledge of EP and LGE-MRI scans. For example, Shade et al. [49] built models to predict AF recurrence using a small cohort of 32 patients. The highest performance (AUC = 0.82) was achieved by combining imaging features and features extracted from biophysical modeling. However, only a minimal drop in performance was observed when only features from biophysical modeling were used (AUC = 0.81). Moreover, the drop in performance was significant when only imaging features were used (AUC = 0.47). This potentially suggests that integrating existing knowledge into ML-based classification models can reduce the number of labeled samples required for developing an accurate classification model. Similarly, Roney et al. [57] proposed a prediction model based on SVM with principal component analysis (PCA) using imaging biomarkers, biophysical modeling, and non-imaging features, and achieved an AUC of 0.85 ± 0.09. The extraction of biophysical modeling features requires significant domain knowledge in EP. For a prognosis of AF, classification models using biophysical modeling features may achieve satisfactory performance even with small datasets. Shade et al. [49] and Roney et al. [57] used datasets consisting of less than 100 patients, in contrast to the primarily data-driven models that use datasets with hundreds to tens of thousands of patients.

The morphological remodeling of the LA and its substructures, induced by AF, exhibits self-similar properties that can be quantitatively evaluated. For example, Firouznia et al. [54] extracted radiomic features based on the fractal dimension of the LA and its substructures. The highest performance of 0.87 (95% CI, 0.82–0.93) was achieved using a random forest model that incorporated radiomic features extracted from the LA and all its substructures, along with non-imaging features. This model outperformed the models that only included subsets of these features. Hwang et al. [53] proposed a method based on a CNN to predict AF recurrence. Curved M-mode images of global strain and global strain rate were generated from postprocedural TTE. When using images of global strain and global strain rate from the four-chamber view, the DL-based prediction model achieved the highest performance (AUC = 0.861), outperforming the ML-based prediction model, which utilized a combination of handcrafted features. Notably, the TTEs were acquired post-ablation, which differs from other publications predicting AF recurrence using images acquired pre-ablation. Images acquired post-ablation contain relevant information about the ablation and can potentially be more predictive.

Various approaches have been used for post-ablation patient follow-up, which introduces challenges in the prognosis of AF. While cardiac implantable electronic devices offer the most accurate approach for follow-up due to their ability to provide continuous and remote monitoring of heart rhythm, they are not offered to every patient with AF who has undergone catheter ablation. The general approach includes routine check-ups at specific time points post-ablation, along with additional examinations for symptoms, using ECG or Holter monitoring. A 3-month blanking period is typically considered when predicting AF recurrence after catheter ablation, but the timing of scheduled routine examinations varies across publications. Table 5 provides a summary of the follow-up approaches used in the publications. Furthermore, Yang et al. [58] reported the prescription of antiarrhythmic drugs for 8 weeks post-ablation, which was not reported by other authors and can potentially introduce bias to the observed outcome.

In summary, AI-based methods can be used for the prediction of incident AF in different populations, as well as for the prediction of incident HF, a complication of AF. Furthermore, outcomes after rhythm control procedures, including electrical cardioversion and catheter ablation, can be predicted with AI. However, a lack of consistency in follow-up methods was observed in the selected publications, suggesting large, open-source datasets with standardized follow-up strategies are desired for constructing more robust and generalizable AI models.

## 4. Future Directions

Despite the promising applications of AI in image-guided care of AF, there are still challenges to overcome and opportunities for improvement. In this section, we discuss the future directions of AI research for image-guided care of AF. Three aspects are covered: utilizing unlabeled datasets and improving model generalizability (Section 4.1), building better AI models with cutting-edge computational methods and imaging modalities (Section 4.2), and boosting the clinical applicability of AI models (Section 4.3).

### 4.1. Unlabeled Datasets and Generalizability

The current models for both segmentation and classification in image-assisted care of AF are mostly developed through supervised learning, which requires labeled datasets. However, the majority of medical images are unlabeled [87], which hinders their use. Creating labeled datasets in medical imaging demands significant resources and is costly to execute on a large scale [87]. Additionally, the process of creating some labels may introduce bias or inconsistency, as observed in the various approaches used for patient follow-up post-ablation.

To utilize the vast number of unlabeled datasets of medical images, novel approaches for developing AI models that are not fully supervised have been proposed. These approaches include self-supervised learning and weakly supervised learning. Self-supervised learning leverages datasets with a large portion of unlabeled samples and a small portion of expert-labeled samples. The unlabeled samples are used to create a pretext task, wherein an AI model is pre-trained. For example, a model for cardiac chamber segmentation can be pre-trained by predicting anatomical positions [88], which can be automatically defined. By pre-training the segmentation model, it learns the underlying structure of the data. A model pre-trained is known as a featurizer, and when later trained for the downstream task with a small, labeled subset using a fully supervised approach, it can potentially achieve equivalent performance to AI models trained directly using a fully supervised approach with large, labeled datasets [87]. On the other hand, weakly supervised learning trains AI models with weak labels using a fully supervised approach. Compared to strong labels, which are used for regular supervised learning, weak labels require significantly fewer human resources to create. For example, segmentation requires pixel-wise segmentation maps as the strong labels, which are difficult to create on a large scale. Weak labels for segmentation can be points [89], scribbles [90], and bounding boxes [91]. A special type of weak label for segmentation is pseudo segmentation maps, which can be generated by gradient-weighted class activation mapping (Grad-CAM) [92] heatmaps resulting from DL-based image classification tasks [93]. Both self-supervised learning and weakly supervised learning require less human input compared with supervised learning to create labeled datasets and provide alternative solutions to overcome the scarcity of labeled data.

Given the challenges associated with creating large, labeled datasets, most of the publications reviewed relied on a single-institutional dataset for model development. The 2018 LASC dataset [13], which is currently the largest open-source dataset for LA segmentation on LGE-MRI, consists solely of patients enrolled at the University of Utah [94,95]. Typically, a portion of the dataset is held back from the model until model testing. However, if the model is not tested on a completely independent dataset, there could be potential issues with the generalizability of the model.

Models that are highly generalizable are typically evaluated on a completely independent dataset, for example, from a different population [96]. When such ideal settings are not available, splitting the dataset based on a specific time point [52] can serve as a suboptimal approach, in comparison to randomly assigning available samples into training and testing sets. Using time-split datasets can address the issue of generalizability to some extent, as it mimics the process of developing and clinically adopting AI models. However, this approach does not guarantee that the developed model will generalize well to different populations. Therefore, we encourage the creation of large, multi-institutional, open-source datasets, ideally derived from diverse patient populations and on equipment from different manufacturers. Importantly, domain experts should establish the ground truth labels using the same criteria. For example, the ground truth segmentation maps should be created using the same definitions to ensure the structures included in the labels created by different experts are consistent. Similarly, labels created for classification should ideally be acquired using the same approach for diagnosis or outcome assessments.

### 4.2. Cutting-Edge Methods and Modalities

Convolution layers have been instrumental in the success of CNN for image-related tasks [28]. CNN is currently used as the de facto model for segmentation and classification tasks based on DL [29]. Using local receptive fields, CNN-based models can effectively extract the correlation between adjacent pixels and learn the important imaging features at local scales. While CNNs are increasingly used to capture multiscale imaging features, particularly at a global level [24], this approach can result in a loss of information when local receptive fields are applied on a larger scale. Recently, vision transformer (ViT) [97] has gained traction in the field of medical imaging. ViT was inspired by the advances in natural language processing [40]. By splitting an image into multiple patches of sub-images and applying flattening operators, ViT can effectively extract the correlation between non-adjacent pixels and learn global imaging features without losing information due to degrading spatial resolution. Hybrid CNN-ViT architectures [27,32] have been proposed, which integrate attention blocks [40] into CNN-based structures. The potential of these hybrid models to outperform purely CNN-based models, especially for segmentation tasks [98,99], has been demonstrated. Furthermore, pre-training of ViT-based models on large-scale datasets is necessary to learn the underlying structure of data [97], and self-supervised learning can facilitate and enhance this process. Although the applications of ViT in image-guided care of AF are currently limited, they show promising potential for the future.

TTE, CT, and MRI are the cardiovascular imaging modalities that patients with AF might undergo to monitor the condition [4]. While the use of nuclear imaging modalities is less common, recent studies have indicated that positron emission tomography (PET)-CT could have a valuable role in detecting local inflammation in the atria in AF patients [100], as well as in assessing AF severity and predicting the success of ablation procedures [101]. In addition, electroanatomic mapping (EAM) is a novel modality that can provide simultaneous information on the anatomy and EP of the heart, creating a surface map of the endocardial surface of the LA. EAM is commonly used in clinical practice to visually guide catheter ablation procedures [102]. Both PET-CT and EAM provide multimodal information, making them complex forms of data that are well-suited for AI processing. Despite the demonstrated benefits of using AI in processing PET-CT [103], the integration of AI with EAM for the clinical care of AF [104] remains relatively unexplored. Therefore, there are enormous opportunities for the application of AI in processing PET-CT and EAM to improve the clinical care of patients with AF.

### 4.3. Clinical Applicability

While AI models have demonstrated potential in enhancing image-guided care for AF, their adoption in clinical practice remains limited. This is partly due to clinicians’ lack of full confidence in these models. To earn complete trust of clinicians, an AI model should be capable of the following:

- Consistently achieving the stated level of performance for every new sample.
- Providing outputs that clinicians can comprehend and interpret.

Ensuring quality control (QC) is crucial to detect when the AI model fails. When performing segmentation tasks, overlaying segmentation maps on the input images and visually inspecting them is a common QC method. However, this approach becomes impractical for clinicians when handling volumetric images on a large scale, as it necessitates individual inspection of each output slice. To streamline the process, a fully automated pipeline that automates both segmentation and subsequent QC is required. As demonstrated by Abdulkareem et al. [15], this can be achieved by implementing a framework for automated QC, such as reverse classification accuracy [105].

In the case of segmentation models, explainability is typically less of a concern as the output can be visualized and easily interpreted. However, for classification models, explainability becomes a critical factor. ML models that involve feature engineering are generally more explainable as the features contributing to the model’s decision-making process can be identified. In contrast, DL models, despite their potential for higher accuracy, often operate in a “black-box” manner, making their decision-making process less transparent and harder to explain.

**Figure 8:**
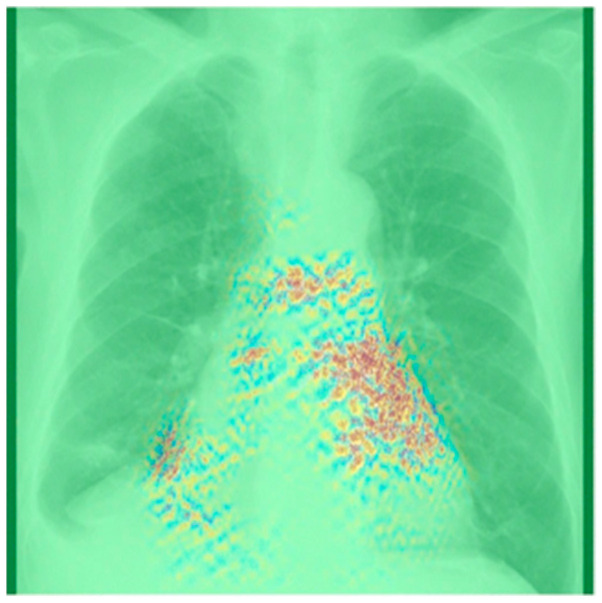
A composite Grad-CAM and guided backpropagation image superimposed on a chest radiograph. The primary region of interest is located in the upper left section of the heart shadow, consistent with the left atrium region. Using Grad-CAM visualization method improves the explainability of the output of deep-learning-based classification model. Figure source: [55].

To enhance the explainability of models, visualization techniques such as Grad-CAM [92] can be employed. Grad-CAM generates a heatmap that highlights the regions that the model focuses on during its decision-making process using the gradients of the target concept, such as the subtle anatomical remodeling associated with AF. Liu et al. [51] used Grad-CAM visualization and identified hotspots in the PVs and the atria. These findings are consistent with clinical observations and imply that the DL model learned features related to the shape and size of the PVs and atria. Similarly, Matsumoto et al. [55] applied Grad-CAM visualization to the true positive predictions, resulting in the regions of interest primarily located in the upper left region of the cardiac shadow, as shown in Figure 8. While Grad-CAM visualization provides a rough visualization of significant regions, it may not be adequate for patients with AF who do not exhibit clear anatomical abnormalities. Hence, more robust methods for explainability, such as the use of DL to efficiently extract interpretable features for classification [106], are required to increase confidence in the diagnosis and prognosis of AF.

## 5. Conclusions

We have thoroughly investigated the current implementations of AI in tasks involving segmentation and classification for the care of AF. Among patients with AF, the LA stands as the central focus. At present, CNN-based methodologies stand at the forefront of achieving automated and consistent LA segmentation. Nevertheless, challenges persist in effectively segmenting intricate LA substructures, including the PVs, the LAA, and the MV.

Numerous AI-driven classification models have been developed to address diverse classification tasks, encompassing AF detection, subtype stratification, and the prediction of both incident and recurrent AF. These diagnostic and prognostic models hold significant potential to augment the precision of image-guided AF care.

Prospective research avenues encompass a broad spectrum, spanning datasets, computational methodologies and imaging modalities, and clinical applicability. Enhancements concerning datasets can be approached from two distinct angles. Firstly, leveraging the substantial reservoir of unlabeled cardiac images can be accomplished through innovative approaches like self-supervised learning and weakly supervised learning. An equally pertinent challenge involves data harmonization. An ideal AI model should seamlessly translate to scans obtained via distinct protocols or machinery from diverse manufacturers. Data harmonization is pivotal to curating a highly variegated dataset for the development of universally applicable models. The acquisition of expansive datasets spanning multiple institutions is pivotal for bolstering both AF segmentation and classification efforts.

Emerging imaging modalities, such as PET-CT and EAM, have been instrumental in characterizing AF. Nonetheless, AI-based analyses of PET-CT or EAM data remain relatively scarce. A comparable trend toward embracing cutting-edge ViT architectures over traditional CNNs for segmentation and classification tasks has emerged within the computer vision domain. However, the potential advantages of ViT in image-guided AF care are yet to be fully harnessed. We firmly believe that explorations into computational methodologies and imaging modalities will usher in transformative advancements for AF care.

Undoubtedly, a medical AI model holds limited utility unless it can be seamlessly integrated into clinical practice. The clinical applicability of such models hinges on their accuracy and interpretability. The development of models geared toward image-guided AF care should not only strive for consistently superior performance but also aspire to heightened transparency in model decision-making processes. Additionally, we anticipate that the strides taken in developing models for AF care can offer a broader roadmap to the EP community, guiding the development of AI applications for rarer yet more severe arrhythmias, such as ventricular tachycardia.

## Figures and Tables

**Table 1:** Summary of publications on artificial intelligence for segmentation included.

Publication (Year) ^1^	Dataset ^2^	Framework	Evaluation Metrics	Highlights
Jin et al. (2018) [19]	*150 ^3^* *LAA*	-	DSC, JSC	Transforming grayscale slices into pseudo color slices improves the spatial resolution of local feature learning. A 3D CRF for post-processing uses the volumetric information to improve 2D segmentation performance from the axial view.
Yang et al. (2018) [16]	100 LA, PVs	TensorFlow	DSC, accuracy, sensitivity, specificity	Applying ConvLSTM to U-net learns the inter-slice correlation from the axial view. Integration of the sequential information with the complementary volumetric information from the coronal and the sagittal views improves 2D segmentation performance from the axial view.
Xiong et al. (2019) [24]	2018 LASC ^4^	TensorFlow	DSC, HD, sensitivity, specificity	Using the unique dual-path architecture with local and global encoders results in highly accurate segmentation of the LA.
Du et al. (2020) [25]	2018 LASC	TensorFlow	DSC, HD	Gradual introduction of the DPM, MSCM, GBMPM, and the deep supervision module to the framework improves segmentation performance in each addition.
Razeghi et al. (2020) [17]	207 ^5^ Multilabel ^6^	TensorFlow	DSC, accuracy, precision, sensitivity, specificity	Using a variant of U-net for automated segmentation of the LA enables reproducible assessment of atrial fibrosis in patients with AF. PV segmentation and MV segmentation result in lower accuracy and higher uncertainty than LA segmentation.
**Borra et al. (2020)** [26] ^7^	2018 LASC	Keras with TensorFlow backend	DSC, HD, sensitivity, specificity	LA segmentation using a 3D variant of U-net outperforms its 2D counterpart. Significant decline in local segmentation accuracy observed in the regions encompassing the PVs.
**Liu et al. (2022)** [27]	2018 LASC	PyTorch	DSC, JSC, HD, ASD	SML structure and uncertainty-guided loss function improve local segmentation accuracy on the fuzzy surface of the LA.
Grigoriadis et al. (2022) [18]	*20 ^8^* *LA, PVs, LAA*	TensorFlow-GPU and Keras library	DSC, HD, ASD, rand error index	Integration of attention blocks with variant of U-net for LA segmentation enhances feature learning.
**Cho et al. (2022)** [14]	118 LA	PyTorch with TensorFlow backend	DSC, precision, sensitivity	Using active learning gradually improves the segmentation performance after each step of human intervention with an initially small, labeled dataset.
Abdulkareem et al. (2022) [15]	*337* *LA*	TensorFlow	DSC	Adoption of a QC mechanism for segmentation enables automated and reproducible estimation of the volume of LA.

^1^ Regular font or **bold font** indicates 2D or **3D** segmentation was performed in the publication. ^2^ The dataset used in each publication. The number of scans and the substructures encompassed in the defined label are provided for publications not using open-source datasets. Scans were acquired in patients with AF unless otherwise stated. *Italic font* or regular font indicates the imaging modality of the dataset was *CECT* or LGE-MRI, respectively. ^3^ Source of scans was not given. ^4^ The 2018 LASC dataset includes a training subset and a testing subset, comprising 100 and 54 LGE-MRI scans, respectively. The labels include the LA endocardium and the LA epicardium. Xiong et al. [24] used both the training and the testing subsets and both the LA endocardium and the LA epicardium labels. The other three publications (Du et al. [25], Borra et al. [26], and Liu et al. [27]) using the 2018 LASC dataset only had access to the training subset and the LA endocardium label. ^5^ Source of scans includes patients with AF (n = 187) and patients without AF (n = 20). ^6^ Labels were defined for the LA, the combined structure of the PVs and the LAA, and the MV separately. ^7^ Both 2D and 3D segmentation were performed. ^8.^ Scans were acquired in patients without AF.

**Table 2:** Important building blocks integrated with U-net architecture for left atrium segmentation.

Building Blocks	Usage and Significance for Segmentation
ConvLSTM	Integrated with U-net to connect the encoder and the decoder for learning the sequential information between adjacent slices from the axial view [16].
Batch Normalization	Applied in each convolutional layer before the activation function so that the segmentation models are less sensitive to the initial parameters, therefore accelerating the training process [15,17,26].
Squeeze and Excitation	An additional block included in each convolutional layer of ResUNet++ to adapt model response according to feature relevance [18].
ASPP	Connects the encoder and the decoder in the ResUNet++ architecture to facilitate multiscale feature learning [18].
Attention	Attention blocks in the decoder of the ResUNet++ architecture enhance focus on the essential region of the input slices [18].
Dropout	Prevents model overfitting so that the developed models are more generalizable to unseen population [15,26].

**Table 3:** Comparison of segmentation performance of the selected publications using the 2018 left atrium segmentation challenge dataset.

Publication (Year)	Architecture	DSC	HD (mm)
Du et al. (2020) [25]	2D framework comprising DPM, MSCM, and GBMPM.	0.94	11.89
Borra et al. (2020) [26]	3D variant of U-net.	0.91	8.34
Liu et al. (2022) [27]	3D network based on V-net with integrated SML structure.	0.92	11.68

**Table 5:** Summary of various assessments used for post-ablation patient follow-up in prognosis of atrial fibrillation.

Publication (Year)	Routine Assessments (Post-Ablation)	Symptomatic Assessments
Shade et al. (2020) [49]	3, 6, and 12 months	Yes
Vinter et al. (2020) [50]	3 months	Yes
Hwang et al. (2020) [53]	1 week; 1, 3, and 6 months; and every 3–6 months	Yes
Firouznia et al. (2021) [54]	3, 6, and 12 months *	Not specified
Roney et al. (2022) [57]	2–4 appointments over 1 year	Not specified
Yang et al. (2022) [58]	Not specified	

* Assessments were performed using integrated clinical assessments and automated patient-reported outcome [86].

## Data Availability

Not applicable.

## Abbreviations

2D	Two-dimensional
3D	Three-dimensional
AF	Atrial fibrillation
AI	Artificial intelligence
ASD	Average surface distance
ASPP	Atrous spatial pyramidal pooling
AUC	Area under the curve
CECT	Contrast-enhanced computed tomography
CI	Confidence interval
CNN	Convolutional neural network
ConvLSTM	Convolutional long short-term memory
CRF	Conditional random field
CT	Computed tomography
DL	Deep learning
DPM	Dual-path module
DSC	Dice similarity coefficient
EAM	Electroanatomic mapping
EAT	Epicardial adipose tissue
ECG	Electrocardiography
EP	Electrophysiology
GBMPM	Gated bidirectional message passing module
Grad-CAM	Gradient-weighted class activation mapping
HD	Hausdorff distance
HF	Heart failure
JSC	Jaccard similarity coefficient
LA	Left atrium
LAA	Left atrial appendage
LASC	Left atrium segmentation challenge
LGE-MRI	Late gadolinium-enhanced magnetic resonance imaging
LV	Left ventricle
LVEF	Left ventricular ejection fraction
ML	Machine learning
MRI	Magnetic resonance imaging
MSCM	Multiscale context-aware module
MV	Mitral valve
PAF	Paroxysmal atrial fibrillation
PCA	Principal component analysis
PeAF	Persistent atrial fibrillation
PET	Positron emission tomography
PV	Pulmonary vein
QC	Quality control
RA	Right atrium
SML	Symmetric multilevel supervision
SVM	Support vector machine
TTE	Transthoracic echocardiography
ViT	Vision transformer
